# Adaptively Optimized Combination of Phased‐Array MR Spectroscopy Data Without Noise Correlation Estimation: A Joint Diagonalization Method

**DOI:** 10.1002/nbm.70371

**Published:** 2026-09-27

**Authors:** Liang Fang, Minjie Wu, Howard J. Aizenstein, Anand Kumar, Shaolin Yang

**Affiliations:** ^1^ Department of Psychiatry University of Pittsburgh Pittsburgh Pennsylvania USA; ^2^ Department of Psychiatry University of Illinois at Chicago Chicago Illinois USA

**Keywords:** coil combination, joint diagonalization, magnetic resonance spectroscopy, phased array

## Abstract

The adaptively optimized combination (AOC) method optimally combines multichannel magnetic resonance spectroscopy (MRS) data by incorporating noise correlations among receive coil elements/channels. These correlations are typically estimated from noise samples extracted from the fully decayed tails of acquired free induction decays (FIDs). To eliminate the need for FID noise tails and the corresponding interchannel noise correlation estimation, we propose a novel coil‐combination method, termed AOC‐JD, that exploits the intrinsic multichannel noise information contained in the differences between individual repetitions and the repetition‐averaged spectroscopic data. The proposed method is based on joint diagonalization (JD) of two correlation matrices constructed from the individual repetitions and the repetition‐averaged multichannel MRS data, thereby circumventing the conventional interchannel noise correlation estimation procedure. The performance of AOC‐JD was evaluated using both computer simulations and in vivo human brain experiments. The results demonstrate that, when multichannel FIDs do not contain valid or sufficiently long noise tails, AOC‐JD significantly improves the signal‐to‐noise ratio (SNR) of the combined spectra compared with the conventional singular value decomposition (SVD) method. Moreover, its performance is comparable to that of the original AOC method, which is optimized to maximize the SNR of the combined spectrum, when applicable. These findings demonstrate that AOC‐JD provides a theoretically grounded and reliable approach for combining multichannel MRS data when FID noise tails are unavailable or insufficient.

## Graphical abstract

A novel method for adaptively optimized combination (AOC) of multichannel magnetic resonance spectroscopy (MRS) data was developed that requires neither noise samples nor estimation of noise correlations across coil elements/channels. The method employs joint diagonalization (JD) of two correlation matrices: one derived from the individual repetitions of the multichannel spectroscopic data and the other from the multichannel spectroscopic data averaged across repetitions. This proposed AOC‐JD method achieves SNR improvement comparable to that of the original AOC method and significantly outperforms the conventional SVD method.

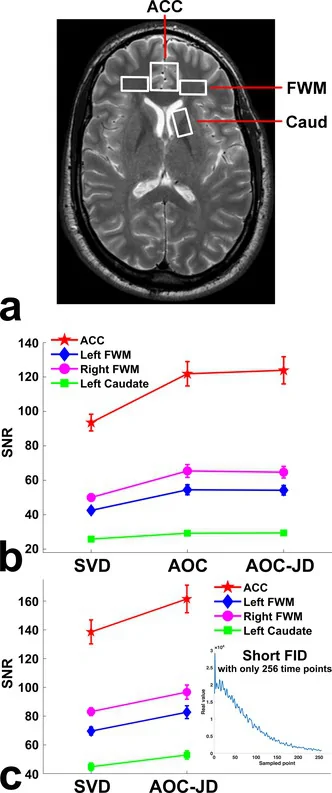

## 1 INTRODUCTION

Magnetic resonance spectroscopy (MRS) provides a noninvasive means to probe in vivo metabolism through quantification of endogenous metabolite concentrations [1, 2, 3]. However, the inherently low MR sensitivity, combined with low metabolite concentrations, results in a relatively low signal‐to‐noise ratio (SNR). This low SNR represents a significant limitation for the clinical applications of MRS [4]. Advances in MRI hardware, including multichannel phased‐array receive coils, have enabled phased‐array MRS to achieve higher SNR through optimized coil‐combination methods [5, 6, 7, 8, 9, 10, 11, 12, 13, 14, 15, 16, 17, 18, 19, 20, 21].

Methods for constructive combination of multichannel MR spectroscopic data differ primarily in the weighting coefficients assigned to individual coil elements/channels and can be broadly classified into two categories. One category determines the weighting coefficients of coil elements/channels without accounting for multichannel noise characteristics [5, 6, 7, 8, 9]. The singular value decomposition (SVD) method [8], a representative method in this category, decomposes a matrix constructed from the received multichannel spectroscopic data to extract the principal component, which serves as the weighting coefficients of coil elements/channels. The other category uses noise samples from the received multichannel spectroscopic data to estimate the weighting coefficients of coil elements/channels [10, 11, 12, 13, 14, 15, 16, 17, 18, 19, 20, 21]. Note that in ^1^H MRS, noise samples are often obtained from the later, decayed portion of the received free induction decays (FIDs). Selecting noise samples from the spectral domain can be problematic because the non‐flat spectral baseline induced by different sources such as macromolecules, lipids, and residual water signal can bias noise samples in the spectral domain. Within this category, the adaptively optimized combination (AOC) method [19], developed by our group, incorporates the inverse of the full noise correlation matrix across coil elements/channels into the weighting coefficients. It effectively suppresses nearly all types of noise, thereby improving the SNR of the combined spectrum in an adaptive and robust manner. The AOC method has been shown to outperform other existing methods [5, 6, 7, 8, 9, 10, 11, 12, 13, 14, 15, 16, 17, 18] in terms of SNR enhancement and preserving the combined spectrum from distortion [19, 20]. However, the AOC method requires sufficient noise samples to accurately estimate the noise correlation among coil elements/channels. This requirement may not be met when noise samples are unavailable or insufficient in the acquired multichannel spectroscopic data. For example, in ultrashort TR and TE ^1^H FID magnetic resonance spectroscopic imaging (FID‐MRSI) [22, 23], the readout duration is significantly reduced by decreasing the number of sampling points and using a shorter dwell time. As a result, the later, decayed portion of the FID, where the noise samples are typically obtained, may not be acquired at all or may be sampled insufficiently. Moreover, noise samples obtained from a separate prescan may not accurately reflect the noise characteristics of the subsequently acquired multichannel spectroscopic data, because multichannel noise may be nonstationary, owing to factors such as subject motion and changes in coil loading [24]. Under these circumstances, the AOC method, which relies on noise correlation estimation, is not feasible for achieving an optimal coil combination. In contrast, the SVD method, which does not utilize noise samples, is commonly applied but results in a suboptimal combination of multichannel spectroscopic data [22]. This raises an important question: Is there a universally applicable coil‐combination method that remains optimal even when noise samples are unavailable or insufficient in the acquired multichannel spectroscopic data?

It has been shown that incorporating multichannel noise characteristics into the weighting coefficients of coil elements/channels is critical for achieving optimal coil combination [19, 20]. In the absence of explicit or pure noise samples that are typically obtained from the later, decayed portion of received FIDs in ^1^H MRS, multichannel noise can alternatively be estimated from the noise components inherently embedded in the acquired multichannel spectroscopic data. In routine MRS applications, an ensemble of multichannel spectroscopic data is typically acquired through repeated measurements [3]. Spectroscopic data from individual coil elements/channels are often averaged across these repetitions prior to coil combination, yielding averaged multichannel spectroscopic data with improved SNR. This SNR improvement arises because metabolite resonance signal adds coherently across repetitions, whereas noise tends to add incoherently and cancel out. Accordingly, compared with the raw multichannel spectroscopic data from individual repetitions, the averaged data across these repetitions preserve the coherent signal component while reducing noise variance in inverse proportion to the number of repetitions [25]. As a result, the difference between the raw and averaged multichannel spectroscopic data contains valuable information about the underlying multichannel noise. However, this noise information has largely been overlooked in MRS, as coil combination is typically performed using only the averaged multichannel spectroscopic data [5, 6, 7, 8, 9, 10, 11, 12, 13, 14, 15, 16, 17, 18, 19, 20, 21]. To fully exploit the underlying multichannel noise for optimal coil combination, it is therefore necessary to use both the raw multichannel spectroscopic data across the repetitions and the corresponding averaged multichannel spectroscopic data.

In the present study, we developed a novel method for adaptively optimized combination of multichannel MRS data that does not require noise correlation estimation. This method employs joint diagonalization (JD) of two correlation matrices: one calculated from the raw multichannel spectroscopic data from individual repetitions and the other from the corresponding multichannel spectroscopic data averaged across repetitions. The performance of the proposed method was evaluated using computer simulations and in vivo human brain experiments conducted on a 3 T MRI scanner with an 8‐channel phased‐array head coil. Compared with the conventional SVD method [8], the proposed method yielded significantly higher SNR in the combined spectrum. Furthermore, compared with the original AOC method [19], it achieved comparable SNR enhancement without requiring noise correlation estimation.

## 2 Methods

### 2.1 Adaptively Optimized Combination of Multichannel MRS via Joint Diagonalization

Individual elements of a phased‐array coil generally exhibit different coil sensitivities and spatial phase shifts owing to their varying positions relative to the voxel of interest (VOI). In addition, the MR spectroscopic signal received from each coil element/channel is inevitably contaminated by noise. Accordingly, the MR spectroscopic signal $y_{i}\left(t\right)$ received from the i th coil element/channel can be modeled as

(1) $$y_{i}\left(t\right)=b_{i}s\left(t\right)+n_{i}\left(t\right)=x_{i}\left(t\right)+n_{i}\left(t\right)$$

where $s\left(t\right)$ denotes the metabolite resonance signal originating from the VOI; $b_{i}=\left|b_{i}\right|e^{j\theta_{i}}$ is a complex coefficient, with $\left|b_{i}\right|$ and $\theta_{i}$ corresponding to the coil sensitivity and spatial phase shift of the i th coil element/channel, respectively; $x_{i}\left(t\right)=b_{i}s\left(t\right)$ denotes the signal received from the i th coil element/channel; and $n_{i}\left(t\right)$ represents the noise on the i th coil element/channel. Given a sampling frequency $f_{s}$, the sampled spectroscopic data from the i th coil element/channel can be written as

(2) $$y_{i}\left(p\right)=b_{i}s\left(p\right)+n_{i}\left(p\right)=x_{i}\left(p\right)+n_{i}\left(p\right)$$

where $p=f_{s}t$ indexes the discrete samples. An ensemble of multichannel spectroscopic data typically consists of multiple repetitions, which can be expressed in matrix form by concatenating the repeated acquisitions along the time dimension as follows:

(3) Y=X+N

where Y, X, and N are M×PL matrices, with M representing the number of coil elements/channels, P the number of sampled spectroscopic data points per repetition, and L the number of repetitions; X and N denote the metabolite resonance signal and the noise, respectively. The weighted linear summation of the multichannel spectroscopic data Y is given by

(4) $$z=w^{H}Y$$

where w is an M×1 complex‐valued weighting vector of coil elements/channels, and H denotes the conjugate transpose. The goal here is to determine an optimal weighting vector w that maximizes the SNR of the combined spectroscopic data z.

According to Equations (3) and (4), the SNR of the combined spectroscopic data z can be calculated as [26].

(5) $$\mathrm{SNR}=\frac{w^{\text{H}}R_{X}w}{w^{\text{H}}R_{N}w}$$

where $R_{X}=XX^{H}$ is the signal correlation matrix, and $R_{N}=NN^{H}$ is the noise correlation matrix. To maximize the SNR, the following constrained optimization problem is formulated

(6) $$\begin{array}{l} \max\;\begin{array}{cc} & \mathrm{SNR} \end{array}=\frac{w^{H}R_{X}w}{w^{H}R_{N}w}\\ \text{subject}\begin{array}{cc} \;\text{to} & w^{H}R_{N}w=1 \end{array} \end{array}$$

where the noise power is normalized. The method of Lagrange multipliers [27], a strategy for finding local maxima and minima of a function subject to equality constraints, can be employed to convert the constrained problem into an unconstrained one as follows:

(7) $$L\left(w\right)=w^{H}R_{X}w+\lambda \;\left(1-w^{H}R_{N}w\right)$$

Taking the derivative of $L\left(w\right)$ with respect to $w^{*}$ (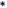 denotes conjugate) yields

(8) $$\frac{\partial L\left(w\right)}{\partial w^{*}}=R_{X}w-\lambda \;R_{N}w$$

Setting the right‐hand side of Equation (8) to the zero vector yields

(9) $$R_{X}w=\lambda \;R_{N}w$$

This shows that the weighting vector w and the parameter λ are the generalized eigenvector and generalized eigenvalue, respectively, of the matrix pencil $\left(R_{X},R_{N}\right)$. We can further derive

(10) $$\lambda =\frac{w^{H}R_{X}w}{w^{H}R_{N}w}=\mathrm{SNR}$$

It indicates that the generalized eigenvalue of the matrix pencil $\left(R_{X},R_{N}\right)$ represents the SNR of the combined spectroscopic data. Therefore, in order to maximize the SNR, we need to find the maximum generalized eigenvalue. In this way, the generalized eigenvector that corresponds to the maximum generalized eigenvalue represents the optimal weighting vector of coil elements/channels.

Note that if the noise from individual coil elements/channels is uncorrelated and identically Gaussian distributed, the noise correlation matrix $R_{N}$ reduces to the identity matrix, that is,

(11) $$R_{N}=I_{M}$$

where $I_{M}$ is an M×M identity matrix. Assuming statistical independence between the metabolite resonance signal and the noise, the correlation matrix of the multichannel spectroscopic data Y is given by

(12) $$R_{Y}=YY^{H}=R_{X}+R_{N}$$

By substituting Equations (11) and (12) into Equation (9), we obtain

(13) $$R_{Y}w=\left(\lambda +1\right)w$$

It shows that, under the idealized condition of uncorrelated, identically distributed Gaussian noise across coil elements/channels, the optimal weighting vector reduces to the eigenvector associated with the largest eigenvalue of the correlation matrix of the multichannel spectroscopic data, rendering the proposed method equivalent to the conventional SVD method [8].

In fact, the aforementioned strategy for optimal coil combination is consistent with the AOC method [19], as both address the same constrained maximization problem described in Equation (6). In the AOC method, the optimal weighting vector of coil elements/channels is derived from estimates of coil sensitivities, spatial phase shifts, and intercoil noise correlations. Consequently, the AOC method becomes impractical when reliable estimates of the intercoil noise correlations are unavailable. In contrast, in the present study, we achieve adaptively optimized combination (AOC) via joint diagonalization (JD) without requiring the knowledge of noise correlation between coil elements/channels. We therefore refer to this new method as “AOC‐JD.”

Assuming that $\lambda_{1},\lambda_{2},\cdots ,\lambda_{M}$ and $w_{1},w_{2},\cdots ,w_{M}$ are the generalized eigenvalues and generalized eigenvectors, respectively, of the matrix pencil $\left(R_{X},R_{N}\right)$, Equation (9) can be rewritten as

(14) $$R_{X}W=R_{N}W\Lambda_{1}$$

where $W=\left[\begin{array}{cccc} w_{1} & w_{2} & \cdots & w_{M} \end{array}\right]$ and $\Lambda_{1}=\mathrm{diag}\left(\lambda_{1},\lambda_{2},\cdots ,\lambda_{M}\right)$ are the generalized eigenvector matrix and generalized eigenvalue matrix, respectively, of the matrix pencil $\left(R_{X},R_{N}\right)$. Based on the relationship between generalized eigendecomposition and joint diagonalization (see Appendix A), the two Hermitian matrices $R_{X}$ and $R_{N}$ can be simultaneously diagonalized as

(15) $$W^{H}R_{N}W=I_{M}$$

(16) $$W^{H}R_{X}W=\Lambda_{1}$$

In addition, it is known that the matrix W, which simultaneously diagonalizes the two Hermitian matrices $R_{X}$ and $R_{N}$, also diagonalizes any linear combination of the two matrices.

We further consider the multichannel spectroscopic data averaged across multiple repetitions, which can be formulated as follows:

(17) $$Z=\frac{1}{L}YF=\bar{X}+\bar{N}$$

where the PL×P matrix F is constructed by vertically concatenating L identity matrices of size of P×P; $\bar{X}$ and $\bar{N}$ are M×P matrices representing the averaged metabolite resonance signal and noise, respectively. Assuming that the averaging operation F attenuates the noise by a factor of $g_{N}$ and enhances the metabolite resonance signal by a factor of $g_{X}$, but leaves the spatial correlation structure within each matrix unchanged, we have

(18) $$R_{\bar{X}}=\bar{X}{\bar{X}}^{H}=g_{X}^{2}R_{X}$$

(19) $$R_{\bar{N}}=\bar{N}{\bar{N}}^{H}=g_{N}^{2}R_{N}$$

where $R_{\bar{X}}$ and $R_{\bar{N}}$ denote the correlation matrices of the averaged metabolite resonance signal and noise, respectively. Analogous to Equation (12), the correlation matrix of the averaged multichannel spectroscopic data can be expressed as

(20) $$R_{Z}=ZZ^{H}=g_{X}^{2}R_{X}+g_{N}^{2}R_{N}$$

Equations (12) and (20) show that the correlation matrices $R_{Y}$ and $R_{Z}$ are both linear combinations of the matrices $R_{X}$ and $R_{N}$. Therefore, the generalized eigenvector matrix W of the matrix pencil $\left(R_{X},R_{N}\right)$, which simultaneously diagonalizes $R_{X}$ and $R_{N}$, also diagonalizes any linear combination of these two matrices, including the correlation matrices $R_{Y}$ and $R_{Z}$, that is,

(21) $$W^{H}R_{Y}W=\Lambda_{Y}$$

(22) $$W^{H}R_{Z}W=\Lambda_{Z}$$

where $\Lambda_{Y}=\mathrm{diag}\left(\sigma_{Y_{1}}^{2},\sigma_{Y_{2}}^{2},\cdots ,\sigma_{Y_{M}}^{2}\right)$ and $\Lambda_{Z}=\mathrm{diag}\left(\sigma_{Z_{1}}^{2},\sigma_{Z_{2}}^{2},\cdots ,\sigma_{Z_{M}}^{2}\right)$ are diagonal matrices, and $\sigma_{Y_{i}}^{2}$ and $\sigma_{Z_{i}}^{2}$ denote the powers of the raw and averaged multichannel spectroscopic data, respectively. This allows us to alternatively derive the generalized eigenvector matrix W of the matrix pencil $\left(R_{X},R_{N}\right)$ from the correlation matrices $R_{Y}$ and $R_{Z}$, rather than from the correlation matrices $R_{X}$ and $R_{N}$. To the best of our knowledge, this strategy has not been previously reported in the literature.

Based on the relationship between generalized eigendecomposition and joint diagonalization (see Appendix A), the matrix W, which simultaneously diagonalizes the two matrices $R_{Y}$ and $R_{Z}$, can be obtained as the generalized eigenvector matrix of the matrix pencil $\left(R_{Z},R_{Y}\right)$, that is,

(23) $$R_{Z}W=R_{Y}W\Lambda_{2}$$

where $\Lambda_{2}=\mathrm{diag}\left(\frac{\sigma_{Z_{1}}^{2}}{\sigma_{Y_{1}}^{2}},\frac{\sigma_{Z_{2}}^{2}}{\sigma_{Y_{2}}^{2}},\cdots ,\frac{\sigma_{Z_{M}}^{2}}{\sigma_{Y_{M}}^{2}}\right)$ is the generalized eigenvalue matrix, with each generalized eigenvalue $\frac{\sigma_{Z_{i}}^{2}}{\sigma_{Y_{i}}^{2}}$ representing the power ratio of the multichannel spectroscopic data averaged across multiple repetitions to the raw repetitions of multichannel spectroscopic data. Equations (14) and (23) demonstrate that the generalized eigenvector of the matrix pencil $\left(R_{Z},R_{Y}\right)$, which maximizes the power ratio $\frac{\sigma_{Z_{i}}^{2}}{\sigma_{Y_{i}}^{2}}$, also maximizes the power ratio of signal to noise. Therefore, the optimal weighting vector of coil elements/channels $w_{o}$ can be obtained as the generalized eigenvector associated with the largest generalized eigenvalue of the matrix pencil $\left(R_{Z},R_{Y}\right)$. Finally, the combined MR spectroscopic dataset obtained using the proposed AOC‐JD method is given by

(24) $$s=w_{o}^{H}Z=w_{o}^{H}YF$$

The pseudocode for the complete implementation of the AOC‐JD method is provided in Appendix B.

### 2.2 Simulation‐Based Evaluation of AOC‐JD

A high‐SNR FID with a relatively long decayed tail (hereafter referred to as the “reference spectroscopic data”) was acquired from a voxel encompassing the rostral anterior cingulate cortex (ACC) of a participant (see the in vivo spectroscopic data acquisition in the following subsection). The reference spectroscopic data were used to generate a set of simulated 8‐channel spectroscopic data. Each 8‐channel dataset, comprising 128 repetitions, was synthesized by scaling and phase‐shifting the reference spectroscopic data according to predefined amplitudes and phases assigned to the individual coil elements/channels, followed by repeated addition of simulated stochastic multichannel noise. The simulated multichannel noise matrix was modeled as

(25) $$N=\left(A+B\right)\;\tilde{N}$$

where A is an M×M matrix whose nonzero off‐diagonal elements represent the noise correlation between coil elements/channels, B is an M×M diagonal matrix whose diagonal elements represent the simulated noise variances, and $\tilde{N}$ is an M×P matrix containing M uncorrelated, identically distributed Gaussian noises, each consisting of P sampled points. Two multichannel noise scenarios were simulated: (1) uncorrelated noise with identical variances across coil elements/channels (A=0,$B=I_{M}$), and (2) correlated noise with unequal variances across coil elements/channels (A≠0,$B\neq I_{M}$). Accordingly, two 8‐channel spectroscopic datasets with distinct noise characteristics were generated. Each dataset was then combined using three different methods: the conventional SVD method [8], the AOC method [19], and the proposed AOC‐JD method. For the SVD and AOC methods, the weighting coefficients of coil elements/channels were estimated solely from the multichannel spectroscopic data averaged across repetitions. In contrast, the proposed AOC‐JD method estimated the weighting coefficients using both the raw individual repetitions of multichannel spectroscopic data and the multichannel spectroscopic data averaged across repetitions.

A third 8‐channel spectroscopic dataset, comprising 128 repetitions and correlated noise with unequal variances across coil elements/channels, was synthesized using a resampled version of the reference spectroscopic data with a substantially shorter decay tail. The resampled reference spectroscopic data were generated by decimating the original reference spectroscopic data by a factor of 2 and retaining the first 512 complex data points. This configuration mimics spectroscopic data acquired with an echo‐planar readout in echo‐planar spectroscopic imaging (EPSI) [28, 29], where the spectral bandwidth and the number of readout gradient lobes are limited. The synthesized dataset was then combined using the conventional SVD method [8] and the proposed AOC‐JD method. The AOC method [19] was not applicable in this scenario because accurate estimation of noise correlation between coil elements/channels requires a sufficient number of noise samples from the long FID decay tail, which were unavailable in the shortened dataset.

To quantitatively evaluate the performance of the different coil‐combination methods, the SNR of the combined spectrum was calculated as follows:

(26) SNR=a/σ

where a denotes the amplitude of the *N*‐acetylaspartate (NAA) peak at 2.01 ppm, and σ denotes the noise level, estimated as the root mean square of 256 data points within a spectral region free of metabolite resonances (e.g., from −1.176 to −3.129 ppm).

### 2.3 Evaluation of AOC‐JD in Human Brain Experiments

Human brain experiments were conducted in 12 healthy participants to evaluate the performance of the proposed AOC‐JD method. Written informed consent was obtained from all participants under a protocol approved by the Institutional Review Board. ^1^H‐MRS scans were performed on a Philips Achieva 3 T MRI scanner (Philips Medical Systems, Best, the Netherlands) using an 8‐channel phased‐array head coil (SENSE‐Head‐8). Single‐voxel spectra were acquired using a PRESS sequence with the following parameters: repetition time (TR)/echo time (TE) = 3000/35 ms, 2048 complex data points, spectral bandwidth = 2000 Hz, 128 signal averages with 16‐step phase cycling, and an unsuppressed water acquisition at the beginning for signal scaling and eddy‐current correction. Spectroscopic voxels were placed in the rostral ACC (2 × 2 × 2 cm^3^), the left and right dorsolateral frontal white matter (FWM; 2 × 1 × 2 cm^3^), and a subcortical region encompassing the head of the left caudate nucleus (Caud; 1 × 2 × 2 cm^3^) in each participant (see Figure 4a).

The raw multichannel in vivo spectroscopic data were processed offline in MATLAB (MathWorks, Natick, MA, USA). The data were combined using the conventional SVD method [8], the original AOC method [19], and the proposed AOC‐JD method. The performance of each coil‐combination method was subsequently evaluated using the SNR metric described above.

Statistical analyses were performed on the MRS data acquired from the 12 participants. For each participant, differences in SNR among the three methods (SVD, AOC, and AOC‐JD) were calculated within participant for each region (ACC, left FWM, right FWM, and left Caud). Summary statistics are presented as mean, standard deviation (SD), and median for each method and region. Pairwise comparisons between methods (AOC vs. SVD, AOC‐JD vs. SVD, and AOC‐JD vs. AOC) were performed using the Wilcoxon signed‐rank test, and the corresponding percentage increase in SNR from one method to the other method and the *p*‐values were reported for each region.

## 3 Results

### 3.1 Simulated Experiments

Figure 1 compares the performance of the proposed AOC‐JD method with that of the conventional SVD method and the original AOC method for combining simulated multichannel FIDs with relatively long decayed tails in two multichannel noise scenarios. Figure 1a shows a high‐SNR FID with a relatively long decayed tail, which served as the basis for generating the two sets of simulated multichannel spectroscopic data with distinct noise characteristics. Figure 1b,c shows the results in the presence of uncorrelated noise with identical variances across coil elements/channels. Figure 1b shows the noise correlation matrices calculated from individual repetitions and from the multichannel spectroscopic data averaged across repetitions. The off‐diagonal elements were zeros, indicating the multichannel noise was uncorrelated across coil elements/channels, whereas the identical diagonal elements reflected equal noise variances across coil elements/channels. Notably, the noise correlation matrices derived from individual repetitions of the multichannel spectroscopic data were nearly identical, indicating that the simulated multichannel noise was stationary. Furthermore, the noise correlation matrix obtained from the averaged multichannel spectroscopic data closely matched those obtained from the individual repetitions, demonstrating that the averaging operation preserved the noise correlation structure across coil elements/channels.

**FIGURE 1:**
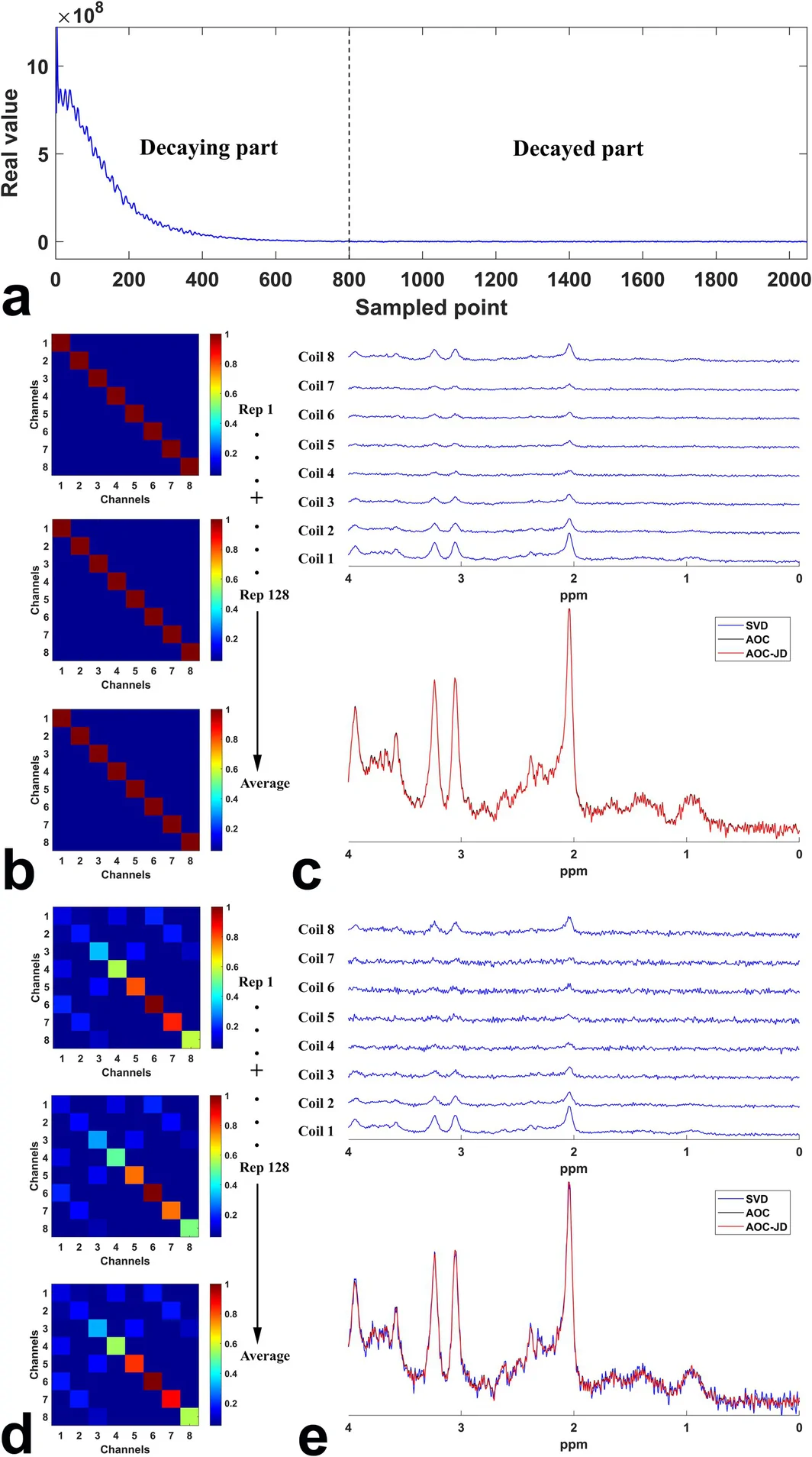
Performance comparison of the proposed AOC‐JD method with the conventional SVD method and the original AOC method for combining simulated multichannel FIDs with relatively long decayed tails in two multichannel noise scenarios: (b,c) uncorrelated noise with identical variances across coil elements/channels and (d,e) correlated noise with unequal variances across coil elements/channels. (a) Reference spectroscopic data used to generate the two simulated multichannel spectroscopic datasets with distinct noise characteristics. For each noise scenario, the noise correlation matrices, calculated from individual repetitions and from the multichannel spectroscopic data averaged across repetitions are shown, together with the averaged spectra from the eight coil elements/channels and the combined spectra obtained using the AOC‐JD, SVD, and AOC methods. Notably, the averaging operation preserved the noise correlation structure across coil elements/channels. The proposed AOC‐JD method achieved SNR performance comparable to that of the AOC method in both multichannel noise scenarios and outperformed the SVD method under correlated noise with unequal variances across coil elements/channels.

Figure 1c displays the averaged spectra from the eight coil elements/channels together with the combined spectra obtained using the three methods. The averaged spectra from individual coil elements/channels exhibited considerable variation in coil sensitivities. The combined spectra obtained using the proposed AOC‐JD method, the SVD method, and the original AOC method were nearly identical, confirming that the three methods perform equivalently in the idealized scenario of uncorrelated, identically distributed Gaussian noise across coil elements/channels. Figure 1d,e presents the results in the scenario of correlated noise with unequal variances across coil elements/channels. Figure 1d shows the corresponding noise correlation matrices calculated from individual repetitions and from the multichannel spectroscopic data averaged across repetitions. The nonzero off‐diagonal elements indicated noise correlation between coil elements/channels, whereas the varying diagonal elements reflected unequal noise variances across coil elements/channels. Notably, the noise correlation matrices derived from individual repetitions and from the multichannel spectroscopic data averaged across repetitions were highly consistent, again demonstrating that the averaging operation preserved the noise correlation structure across coil elements/channels. Figure 1e shows the averaged spectra from each coil element/channel together with the combined spectra obtained using the three methods. The averaged spectra from individual coil elements/channels exhibited distinct noise characteristics. The combined spectrum obtained using the proposed AOC‐JD method closely matched that obtained using the AOC method, whereas the SVD method yielded a spectrum with visibly lower SNR. Quantitatively, the SNRs of the combined spectra were 67.44 for the AOC‐JD method, 66.65 for the AOC method, and 42.03 for the SVD method, respectively, demonstrating a significant improvement in SNR with the proposed AOC‐JD method over the SVD method.

Figure 2 compares the performance of the proposed AOC‐JD method with that of the conventional SVD method for combining simulated multichannel FIDs with a significantly shorter decayed tail under correlated noise with unequal variances across coil elements/channels. Figure 2a shows a resampled version of the FID in Figure 1a with a significantly shorter decayed tail, which served as the basis for generating the simulated multichannel spectroscopic data.

**FIGURE 2:**
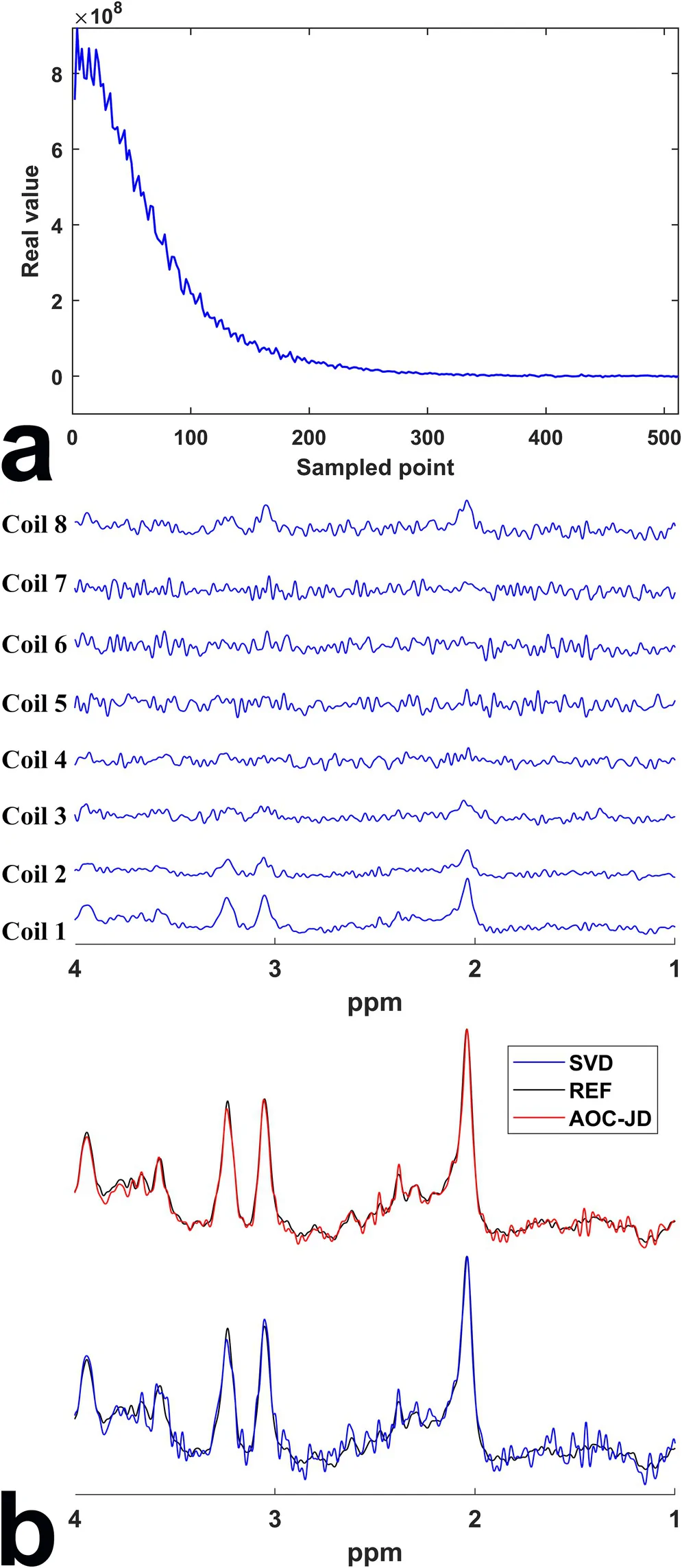
Performance comparison of the proposed AOC‐JD method with the conventional SVD method for combining the simulated multichannel FIDs with a shorter decayed tail in the presence of correlated noise with unequal variances across coil elements/channels. (a) Reference spectroscopic data used as the basis for generating the simulated multichannel spectroscopic data. (b) Averaged spectra from the eight coil elements/channels, together with the combined spectra obtained using the AOC‐JD and SVD methods and the reference spectrum corresponding to the reference spectroscopic data. Notably, the combined spectrum obtained using the proposed AOC‐JD method exhibited lower noise than that obtained using the SVD method.

Figure 2b shows the averaged spectra from the individual coil elements/channels in the simulated correlated‐noise scenario, together with the combined spectra obtained using the AOC‐JD and SVD methods and the reference spectrum corresponding to the spectroscopic data shown in Figure 2a. Compared with the spectra in Figure 1c,e, the averaged spectra from the individual coil elements/channels exhibited reduced spectral resolution and a narrower spectral width because of the shorter acquisition duration and longer dwell time. Notably, the combined spectrum obtained using the proposed AOC‐JD method exhibited substantially lower noise than that obtained using the SVD method, suggesting a potential advantage of the proposed AOC‐JD method for metabolite quantification. Furthermore, the combined spectrum obtained using the AOC‐JD method closely matched the reference spectrum, indicating that the proposed AOC‐JD method preserves spectral fidelity without introducing appreciable distortion. Quantitatively, the combined spectra had SNRs of 18.56 for the AOC‐JD method and 14.78 for the SVD method.

### 3.2 Human Experiments

Figure 3 presents experimental results from multichannel spectroscopic data acquired from a voxel encompassing the right dorsolateral FWM of a participant using an 8‐channel phased‐array head coil. Figure 3a shows the noise correlation matrices calculated from individual repetitions and from the spectroscopic data averaged across repetitions. The diagonal elements of these matrices varied, reflecting differences in noise variances among coil elements/channels. Although the diagonal elements were dominant, several off‐diagonal elements were non‐negligible, indicating noise correlation between coil elements/channels. Importantly, the noise correlation matrix derived from the averaged spectroscopic data closely resembled those derived from the individual repetitions, supporting the assumption that averaging preserves the noise correlation structure across coil elements/channels.

**FIGURE 3:**
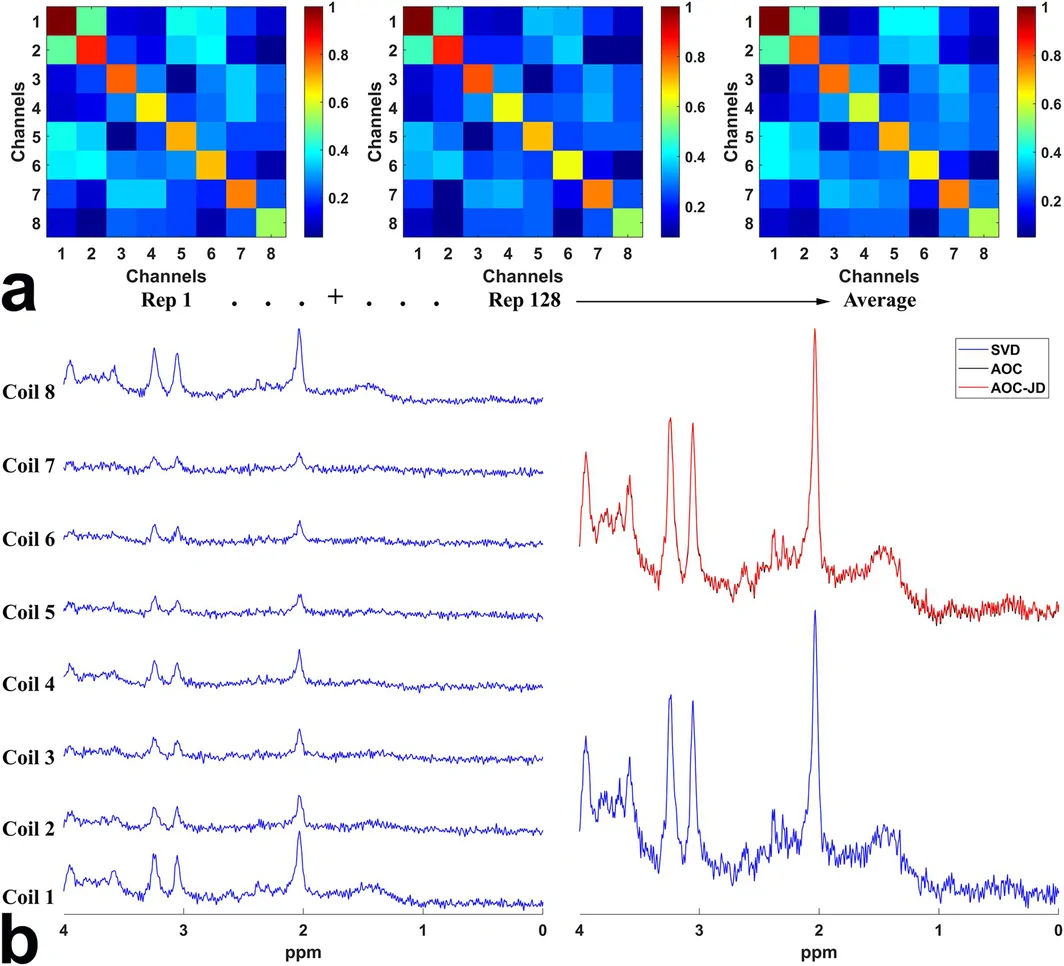
Experimental results from multichannel spectroscopic data acquired from a voxel encompassing the right dorsolateral frontal white matter (FWM) of a participant using an 8‐channel phased‐array head coil. (a) Noise correlation matrices calculated from individual repetitions and from the spectroscopic data averaged across repetitions. (b) Averaged spectra from the eight coil elements/channels, together with the combined spectra obtained using the proposed AOC‐JD method and the SVD and AOC methods. Notably, the averaging operation preserved the noise correlation structure across coil elements/channels. The SNRs of the combined spectra were 63.10 for the proposed AOC‐JD method, 63.34 for the AOC method, and 45.48 for the SVD method.

Figure 3b displays the averaged spectra from the eight coil elements/channels, together with the combined spectra obtained using the proposed AOC‐JD method, the SVD method, and the original AOC method. Although the individual spectra exhibited similar spectral patterns, their amplitudes varied across coil elements/channels because of differences in the spatial positions of the coil elements relative to the VOI. The combined spectrum obtained using the proposed AOC‐JD method closely matched that obtained using the AOC method and exhibited a higher SNR than that obtained using the SVD method. Specifically, the SNRs of the combined spectra were 63.10 for the AOC‐JD method, 63.34 for the AOC method, and 45.48 for the SVD method.

Figure 4 presents the SNRs of the combined spectra acquired from four brain regions in 12 healthy participants. Figure 4a illustrates the locations and sizes of the four voxels, encompassing the rostral ACC, left and right dorsolateral FWM, and left Caud. Figure 4b shows the SNRs of the combined spectra from these voxels obtained using the proposed AOC‐JD method and the SVD and AOC methods applied to the full acquired FIDs. As shown in Figure 4b and Table 1, the AOC and AOC‐JD methods yielded higher SNRs than the SVD method in all regions examined. In the ACC, the mean SNRs were 93.53 ± 16.79 for SVD, 121.87 ± 24.49 for AOC, and 123.88 ± 27.36 for AOC‐JD. The corresponding values were 42.50 ± 6.21, 54.49 ± 10.04, and 54.22 ± 9.60 in the left FWM; 49.98 ± 7.21, 65.44 ± 12.84, and 64.71 ± 11.71 in the right FWM; and 25.85 ± 3.26, 29.27 ± 4.24, and 29.42 ± 4.01 in the left Caud. Wilcoxon signed‐rank tests demonstrated significant differences between SVD and AOC and between SVD and AOC‐JD across all regions (all *p* = 0.001), whereas no significant differences were observed between AOC and AOC‐JD (ACC: *p* = 0.129; left FWM: *p* = 0.622; right FWM: *p* = 0.204; and left Caud: *p* = 0.470). Relative to SVD, AOC increased the SNR by 30.3% in the ACC, 28.2% in the left FWM, 30.9% in the right FWM, and 13.2% in the left Caud. Similarly, the proposed AOC‐JD method increased the SNR by 32.5%, 27.6%, 29.5%, and 13.8% in the ACC, left FWM, right FWM, and left Caud, respectively. Across all four regions, the proposed AOC‐JD method consistently achieved SNRs comparable to those of the AOC method and higher than those of the SVD method.

**FIGURE 4:**
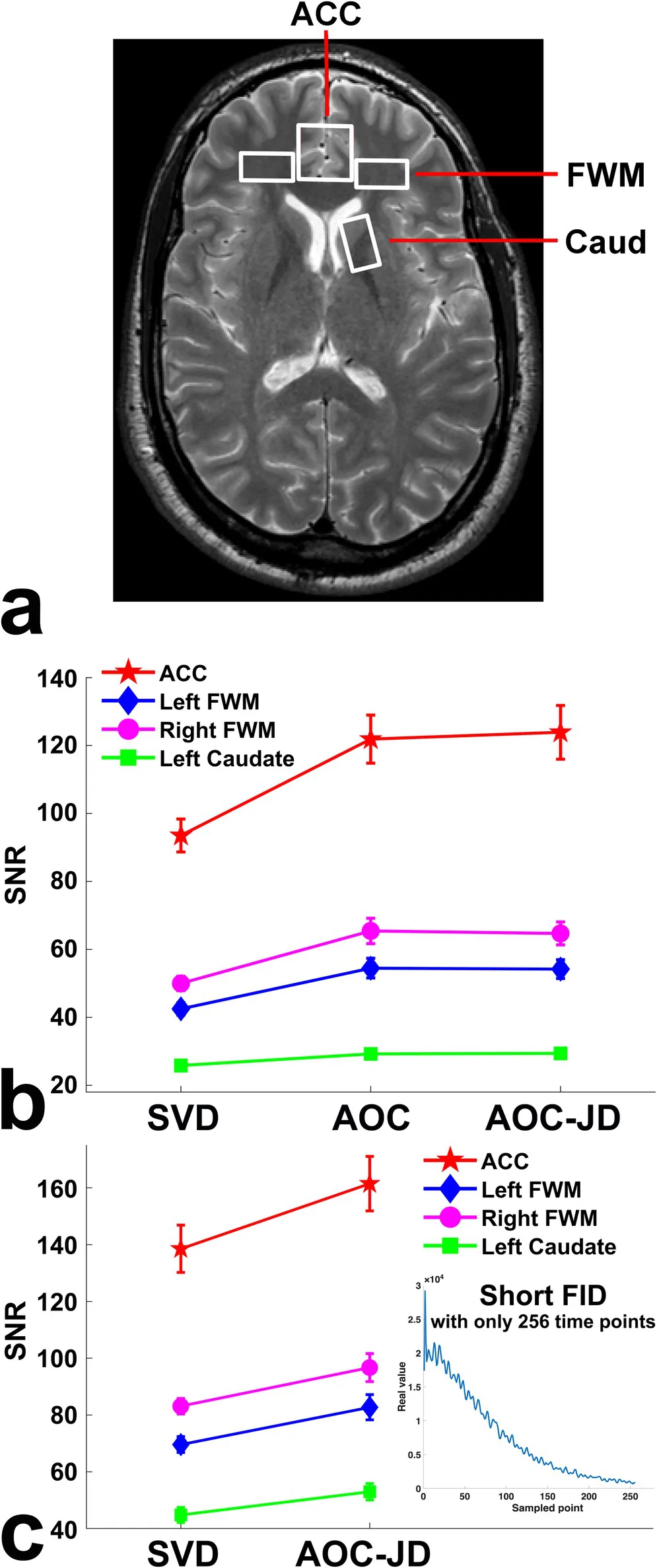
SNRs of the combined spectra acquired from the four brain regions in 12 healthy participants. (a) Locations and sizes of the four voxels encompassing the rostral ACC (2 × 2 × 2 cm^3^), left and right dorsolateral frontal white matter (FWM) (2 × 1 × 2 cm^3^), and left caudate nucleus (Caud) (1 × 2 × 2 cm^3^). (b) SNRs of the combined spectra from the four voxels obtained using the proposed AOC‐JD method and the SVD and AOC methods. Across all four voxels, the proposed AOC‐JD method consistently achieved SNRs comparable to those of the AOC method and higher than those of the SVD method. (c) SNRs of the combined spectra from the four voxels obtained using the proposed AOC‐JD method and the SVD methods applied to substantially shortened FIDs containing only 256 sampled time points. The shortened FIDs encompassed the signal decay until it approached the noise level but excluded the later decayed portion dominated exclusively by noise (see the representative FID from one participant in the inset). The AOC‐JD method consistently yielded significantly higher SNRs than the SVD method across all four regions. Note: Error bars represent the standard error of the mean (SEM).

**TABLE 1:** Comparison of SNR among the SVD, AOC, and AOC‐JD coil‐combination methods.

Region	SVD				AOC				AOC‐JD				*p* (higher SNR ratio)		
	*n*	Mean	SD	Median	*n*	Mean	SD	Median	*n*	Mean	SD	Median	AOC vs. SVD	AOC‐JD vs. SVD	AOC‐JD vs. AOC
**ACC**	12	93.53	16.79	94.42	12	121.87	24.49	120.62	12	123.88	27.36	122.46	0.001 (30.3%)	0.001 (32.5%)	0.129 (1.7%)
**Left FWM**	12	42.50	6.21	43.22	12	54.49	10.04	56.62	12	54.22	9.60	56.47	0.001 (28.2%)	0.001 (27.6%)	0.622 (−0.5%)
**Right FWM**	12	49.98	7.21	46.00	12	65.44	12.84	61.00	12	64.71	11.71	60.29	0.001 (30.9%)	0.001 (29.5%)	0.204 (−1.1%)
**Left Caud**	12	25.85	3.26	25.54	12	29.27	4.24	28.05	12	29.42	4.01	28.55	0.001 (13.2%)	0.001 (13.8%)	0.470 (0.5%)

*Note:* 1. Difference between SNR derived from different methods was calculated within participant. 2. Wilcoxon signed‐rank test was used to assess the difference between methods.

Figure 4c and Table 2 show the SNRs of the combined spectra from the same voxels obtained using the proposed AOC‐JD method and the SVD methods applied to substantially shortened FIDs containing only the first 256 sampled time points. The shortened FIDs encompassed the signal decay until it approached the noise level but excluded the later portion dominated exclusively by noise (see the representative FID from one participant in the inset). The AOC method was not applicable in this shortened‐FID scenario because it relies on the noise tail of the FID to estimate the noise correlation across coil elements/channels. As shown in Table 2, the AOC‐JD method consistently yielded significantly higher SNRs than the SVD method across all four regions (all *p* = 0.001), with SNR increases of 16.6% in the ACC, 18.9% in the left FWM, 16.4% in the right FWM, and 18.2% in the left Caud.

**TABLE 2:** Comparison of SNR between the SVD and AOC‐JD coil‐combination methods for short FIDs.

Region	SVD				AOC‐JD				*p* (higher SNR ratio)
	*n*	Mean	SD	Median	*n*	Mean	SD	median	AOC‐JD vs. SVD
**ACC**	12	138.55	28.89	140.26	12	161.52	33.25	166.18	0.001 (16.6%)
**Left FWM**	12	69.60	9.65	69.58	12	82.74	15.36	81.08	0.001 (18.9%)
**Right FWM**	12	83.11	9.44	85.38	12	96.72	17.07	95.57	0.001 (16.4%)
**Left Caud**	12	44.82	9.17	43.74	12	52.99	9.98	52.24	0.001 (18.2%)

*Note:* 1. Difference between SNR derived from different methods was calculated within participant. 2. Wilcoxon signed‐rank test was used to assess the difference between methods.

## 4 Discussion

We proposed a novel method for adaptively optimized combination of multichannel MR spectroscopic data acquired with a phased‐array coil that does not require explicit estimation of noise correlations between coil elements/channels. The proposed AOC‐JD method employs joint diagonalization of two correlation matrices: one derived from the individual repetitions of multichannel spectroscopic data and the other from the spectroscopic data averaged across repetitions. Compared with the original AOC method [19] and the conventional SVD method [8], the proposed AOC‐JD method achieved performance comparable to that of the AOC method and significantly outperformed the SVD method in enhancing the SNR of the combined spectrum.

The proposed AOC‐JD method is formulated by solving an optimization problem that maximizes the SNR of the combined spectrum, as defined in Equation (6), consistent with the optimization strategy underlying the original AOC method [19]. This theoretical consistency explains why the AOC‐JD method achieved SNR performance comparable to the AOC method, as demonstrated in Figures 1c,e, 3b, and 4b.

In the AOC method, the optimal weighting coefficients for coil elements/channels are directly derived from Equation (9) using estimates of coil sensitivities, spatial phase shifts, and noise correlations between coil elements/channels. In contrast, the proposed AOC‐JD method determines the optimal weighting coefficients through joint diagonalization of two correlation matrices: one calculated from the individual repetitions of multichannel spectroscopic data and the other from the multichannel spectroscopic data averaged across repetitions, as shown in Equations (21) and (22). A key assumption underlying the AOC‐JD method is that averaging preserves the spatial correlation structures of both the noise and the metabolite resonance signal, as described in Equations (18) and (19). In practical MRS acquisitions, this assumption is reasonable when the spatial correlation structures of the multichannel noise and metabolite resonance signal remain stationary across repetitions, as demonstrated in Figures 1b,d and 3a.

Based on the established relationship between joint diagonalization and generalized eigendecomposition (see Appendix A), the AOC‐JD method can be implemented as generalized eigendecomposition of the two correlation matrices (Equation (23)). This formulation can be interpreted as a form of principal component analysis [30] designed to maximize the power ratio of the multichannel spectroscopic data averaged across repetitions to the individual repetitions of multichannel spectroscopic data.

In clinical MRS application, in vivo spectroscopic data are typically acquired with multiple repetitions (e.g., 32, 64, 128, or 256) to compensate for the inherently low SNR. The differences between the individual repetitions and the averaged multichannel spectroscopic data contains valuable information about the underlying multichannel noise. The AOC‐JD method explicitly leverages this information to achieve optimal coil combination. Consequently, it does not require explicit noise samples or knowledge of the noise correlations between coil elements/channels. The proposed AOC‐JD method therefore overcomes a key limitation of the original AOC method, enabling optimized combination of multichannel MRS data even when noise samples are unavailable or insufficient.

This scenario is particularly relevant to fast spectroscopic imaging approaches such as FID‐MRSI, in which the acquired FID is short and the number of repetitions may also be limited. Although single‐voxel MR spectroscopy is typically performed with longer TRs and may therefore permit longer FID acquisitions, obtaining sufficiently clean noise‐only samples or accurately estimating the noise correlation matrix across coil elements/channels can still be challenging in practice. This is especially true when the FID or portions of the spectrum are contaminated by residual water or lipid signals, eddy‐current effects, out‐of‐voxel echoes, baseline distortions, or other artifacts. In addition, physiological and instrumental factors, including subject motion, respiration, pulsation, and scanner instabilities such as fluctuations in receiver gain or coil loading during acquisition, may further complicate noise correlation estimation. Under these conditions, the AOC‐JD method may offer a practical advantage by reducing reliance on explicit noise estimation and thereby limiting the influence of these confounding factors. However, the specific performance and underlying behavior of the method under these different conditions remain to be elucidated through further investigation, as the present study primarily focused on establishing the concept and mathematical foundation of the AOC‐JD method.

Simulation studies demonstrated that the AOC‐JD method consistently achieved SNR performance comparable to that of the original AOC method across different multichannel noise scenarios while outperforming the conventional SVD method, particularly in the presence of correlated noise with unequal variances across coil elements/channels (Figure 1). Importantly, when noise samples were unavailable or insufficient, as illustrated in Figure 2, the AOC method became infeasible because of its reliance on accurate estimation of noise correlations. In contrast, the AOC‐JD method maintained robust performance under correlated noise with unequal variances across coil elements/channels by exploiting intrinsic information about multichannel noise contained in the repeated acquisitions, resulting in improved SNR under these challenging conditions.

In addition to the simulation studies, the AOC‐JD method was evaluated using in vivo human brain data acquired from four voxels at different locations and of different sizes. As shown in Figures 3 and 4 and Tables 1 and 2, the proposed AOC‐JD method consistently achieved SNRs comparable to those of the AOC method and higher than those of the SVD method across all voxels, further demonstrating its robustness and practical utility.

## 5 Conclusions

We introduced a novel method for adaptively optimized combination of multichannel MRS data acquired with phased‐array coils that does not require explicit noise samples or knowledge of noise correlations between coil elements/channels. Both computer simulations and in vivo human brain experiments demonstrated that the proposed AOC‐JD method achieves SNR performance comparable to that of the AOC method while outperforming the conventional SVD method. These findings establish the proposed AOC‐JD method as a theoretically grounded and practically robust approach for multichannel MRS coil combination, particularly when noise samples are unavailable or insufficient in the acquired spectroscopic data.

## Author Contributions

**Liang Fang:** conceptualization, methodology, formal analysis, writing – original draft. **Minjie Wu:** conceptualization, methodology, writing – review and editing. **Howard J. Aizenstein:** conceptualization, investigation, supervision, resources, writing – review and editing. **Anand Kumar:** conceptualization, supervision, resources, project administration, funding acquisition, writing – review and editing. **Shaolin Yang:** conceptualization, methodology, data acquisition and curation, formal analysis, project administration, funding acquisition, writing – original draft, writing – review and editing.

## Conflicts of Interest

The authors declare no conflicts of interest.

## Data Availability

The data that support the findings of this study are available on request from the corresponding author. The data are not publicly available due to privacy or ethical restrictions.

## Appendix A Relationship Between Generalized Eigendecomposition and Joint Diagonalization

To prove that the two Hermitian matrices $R_{1}=\Sigma_{1}\Sigma_{1}^{H}$ and $R_{2}=\Sigma_{2}\Sigma_{2}^{H}$ can be simultaneously diagonalized as follows:

(A1)
$$W^{H}R_{1}W=I$$

(A2)
$$W^{H}R_{2}W=\Lambda$$

where W and Λ are the generalized eigenvector and eigenvalue matrices of the matrix pencil $\left(R_{2},R_{1}\right)$, respectively, that is,

(A3)
$$R_{2}W=R_{1}W\Lambda$$

we can simultaneously diagonalize the two Hermitian matrices $R_{1}$ and $R_{2}$ through the following two steps:

(1) First, we whiten $\Sigma_{1}$ using a linear transformation as follows:

(A4)
$$Y_{1}=\Theta^{-1/2}\Phi^{H}\Sigma_{1}$$

(A5)
$$Y_{2}=\Theta^{-1/2}\Phi^{H}\Sigma_{2}$$

where Θ and Φ are the eigenvalue and eigenvector matrices of $R_{1}$, respectively, satisfying

(A6)
$$R_{1}\Phi =\Phi \Theta$$

(A7)
$$\Phi^{H}\Phi =I$$

As a result, we can obtain

(A8)
$$Y_{1}Y_{1}^{H}=\Theta^{-1/2}\Phi^{H}\Sigma_{1}{\left(\Theta^{-1/2}\Phi^{H}\Sigma_{1}\right)}^{H}=\Theta^{-1/2}\Phi^{H}R_{1}\Phi \Theta^{-1/2}=I$$

(A9)
$$Y_{2}Y_{2}^{H}=\Theta^{-1/2}\Phi^{H}\Sigma_{2}{\left(\Theta^{-1/2}\Phi^{H}\Sigma_{2}\right)}^{H}=\Theta^{-1/2}\Phi^{H}R_{2}\Phi \Theta^{-1/2}=K$$

In general, K is not a diagonal matrix.

(2) Second, we apply an orthonormal transformation to diagonalize K as follows

(A10)
$$Z_{1}=\Psi^{H}Y_{1}$$

(A11)
$$Z_{2}=\Psi^{H}Y_{2}$$

where Ψ is the eigenvector matrix of K, satisfying

(A12)
KΨ=ΨΛ

(A13)
$$\Psi^{H}\Psi =I$$

where Λ is the eigenvalue matrix of K. Thus,

(A14)
$$Z_{1}Z_{1}^{H}=\Psi^{H}Y_{1}{\left(\Psi^{H}Y_{1}\right)}^{H}=\Psi^{H}Y_{1}Y_{1}^{H}\Psi =\Psi^{H}\Theta^{-1/2}\Phi^{H}R_{1}\Phi \Theta^{-1/2}\Psi =I$$

(A15)
$$Z_{2}Z_{2}^{H}=\Psi^{H}Y_{2}{\left(\Psi^{H}Y_{2}\right)}^{H}=\Psi^{H}Y_{2}Y_{2}^{H}\Psi =\Psi^{H}\Theta^{-1/2}\Phi^{H}R_{2}\Phi \Theta^{-1/2}\Psi =\Lambda$$

In this way, the two matrices $R_{1}$ and $R_{2}$ are simultaneously diagonalized. The overall transformation matrix is given by

(A16)
$$W=\Phi \Theta^{-1/2}\Psi$$

By substituting Equation (A9) into Equation (A12), we obtain

(A17)
$$R_{2}\Phi \Theta^{-1/2}\Psi ={\left(\Theta^{-1/2}\Phi^{H}\right)}^{-1}\Psi \Lambda$$

According to Equation (A8), ${\left(\Theta^{-1/2}\Phi^{H}\right)}^{-1}$ can be replaced by $R_{1}\Phi \Theta^{-1/2}$. Thus,

(A18)
$$R_{2}\left(\Phi \Theta^{-1/2}\Psi \right)=R_{1}\left(\Phi \Theta^{-1/2}\Psi \right)\Lambda$$

Equations (A16) and (A18) demonstrate that the transformation matrix $W=\Phi \Theta^{-1/2}\Psi$, which simultaneously diagonalizes the two Hermitian matrices $R_{1}$ and $R_{2}$, also serves as the generalized eigenvector matrix of the matrix pencil $\left(R_{2},R_{1}\right)$.

## Appendix B Pseudocode for Implementing AOC‐JD Coil Combination

Inputs: - MRS_data: raw complex-valued MRS data with dimensions [Nch × Nsamp × Nrep] - Nch = number of coil elements/channels - Nsamp = number of spectroscopic data points sampled per repetition - Nrep = number of repetitionsOutput: - MRS_data_AOC-JD: combined complex-valued MRS data obtained using the AOC-JD method, with dimensions [1 × Nsamp]Algorithm:(1) Average MRS_data across repetitions  MRS_data_mean ← mean MRS_data over the rep dimension  # MRS_data_mean has dimensions [Nch × Nsamp](2) Construct a two-dimensional matrix Y by concatenating all repetitions of MRS_data along the time dimension  Initialize Y as a complex-valued zero matrix with dimensions [Nch × (Nsamp*Nrep)]  For rep = 1 to Nrep:    start_index ← 1 + Nsamp*(rep-1)    end_index  ← Nsamp*rep  Y[:, start_index : end_index] ← MRS_data[:, :, rep]  EndFor  # Y has dimensions [Nch × (Nsamp*Nrep)](3) Compute two covariance-like matrices.   C0 ← Y * (Y)^H    # “full-data” covariance across coils, dimensions [Nch × Nch]  C1 ← MRS_data_mean * (MRS_data_mean)^H    # covariance of the averaged spectroscopic data, dimensions [Nch × Nch]  # ^H denotes the conjugate transpose(4) Generalized eigendecomposition of the two covariance-like matrices C0 and C1  Solve for eigenvectors V and eigenvalues D in:    inv(C0) * C1 * v = λ * v  i.e.,    [V, D] ← eig(C0 \ C1)  # V columns (v) are eigenvectors, D diagonal (λ) are eigenvalues(5) Select the AOC-JD weights  w ← first eigenvector corresponding to the largest eigenvalue:    w ← V[:, 1]  # sorted eigenvalues in descending order(6) Combine the averaged spectroscopic data MRS_data_mean using the AOC-JD weights  MRS_data_AOC-JD ← (w)^H * MRS_data_mean  # Result is the combined complex-valued time-domain spectroscopic data with dimensions [1 × Nsamp]
